# Molecular Insights into the Transmembrane Domain of the Thyrotropin Receptor

**DOI:** 10.1371/journal.pone.0142250

**Published:** 2015-11-06

**Authors:** Vanessa Chantreau, Bruck Taddese, Mathilde Munier, Louis Gourdin, Daniel Henrion, Patrice Rodien, Marie Chabbert

**Affiliations:** 1 UMR CNRS 6214 –INSERM 1083, Laboratory of Integrated Neurovascular and Mitochondrial Biology, University of Angers, Angers, France; 2 Reference Centre for the pathologies of hormonal receptivity, Department of Endocrinology, Centre Hospitalier Universitaire of Angers, Angers, France; University of Rome Tor Vergata, ITALY

## Abstract

The thyrotropin receptor (TSHR) is a G protein-coupled receptor (GPCR) that is member of the leucine-rich repeat subfamily (LGR). In the absence of crystal structure, the success of rational design of ligands targeting the receptor internal cavity depends on the quality of the TSHR models built. In this subfamily, transmembrane helices (TM) 2 and 5 are characterized by the absence of proline compared to most receptors, raising the question of the structural conformation of these helices. To gain insight into the structural properties of these helices, we carried out bioinformatics and experimental studies. Evolutionary analysis of the LGR family revealed a deletion in TM5 but provided no information on TM2. Wild type residues at positions 2.58, 2.59 or 2.60 in TM2 and/or at position 5.50 in TM5 were substituted to proline. Depending on the position of the proline substitution, different effects were observed on membrane expression, glycosylation, constitutive cAMP activity and responses to thyrotropin. Only proline substitution at position 2.59 maintained complex glycosylation and high membrane expression, supporting occurrence of a bulged TM2. The TSHR transmembrane domain was modeled by homology with the orexin 2 receptor, using a protocol that forced the deletion of one residue in the TM5 bulge of the template. The stability of the model was assessed by molecular dynamics simulations. TM5 straightened during the equilibration phase and was stable for the remainder of the simulations. Our data support a structural model of the TSHR transmembrane domain with a bulged TM2 and a straight TM5 that is specific of glycoprotein hormone receptors.

## Introduction

The thyroid stimulating hormone or thyrotropin (TSH) has a major role in the growth and function of the thyroid gland that produces the thyroid hormones T3 (triiodothyronine) and T4 (tetraiodothyronine or thyroxine) [1]. TSH acts by binding to its cognate receptor, TSHR, which is a member of the Leucine-rich repeat subfamily (LGR) of G protein-coupled receptors (GPCRs) [2]. The LGR subfamily includes, among others, TSH, FSH (follitropin) and LH/CG (lutropin/chorionic gonadotropin) receptors. The LGR receptors possess a seven transmembrane helix (TM) domain typical of GPCRs, a large N-terminal domain consisting of a leucine-rich repeat domain (LRR), and a hinge region linking the LRR domain to the transmembrane domain. The LRR domain is the main binding site of the glycoprotein hormones, whereas the hinge and the transmembrane domain are involved in signal transduction.

Low molecular weight ligands targeting TSHR have been developed and can act as agonists or antagonists [3–7]. Contrary to thyrotropin, they bind to the internal cavity of the transmembrane domain. They have therapeutic potential for diseases where the TSHR signal is disturbed, such as Graves’ disease, or in thyroid cancer as an alternative to recombinant human TSH for radioiodine ablation of thyroid remnants or metastases. Currently, crystal structures have been resolved for the ectodomain of TSHR [8] and FSHR [9], but not for their transmembrane domain. Therefore, the rational design of drugs targeting the transmembrane domain of TSHR is highly dependent on the quality of the model(s) used.

The GPCR crystal structures resolved to date illustrate that, although the general fold of the TM domain is conserved within the GPCR superfamily, each receptor displays distinct local structural features, in particular for helical distortions [10]. Helical distortions are frequent in GPCRs and are often stabilized by proline residues [11]. Two main distortions are possible to avoid steric clashes between the pyrole ring of proline and helix backbone: kinks in which proline ring is close to the carbonyl groups at positions -3 and -4, and bulges in which the proline ring is close to the carbonyl groups at positions -4 and -5, because of an additional residue in the helical turn preceding proline [12, 13]. Class A GPCRs possess two highly conserved proline residues in TM6 and TM7. They are part of the CWXP and NPXXY motifs, in TM6 and TM7, respectively, and have an important functional role in activation [11, 14]. Two proline residues in TM2 and TM5 are also present in about 80% of class A GPCRs [15]. The proline residues in TM2 can be found at positions 2.58, 2.59 or 2.60 (Ballesteros’ numbering [16]), with respective weight of 40, 37 and 3% [17] whereas, in TM5, only position 5.50 is observed.

**Fig 1:**
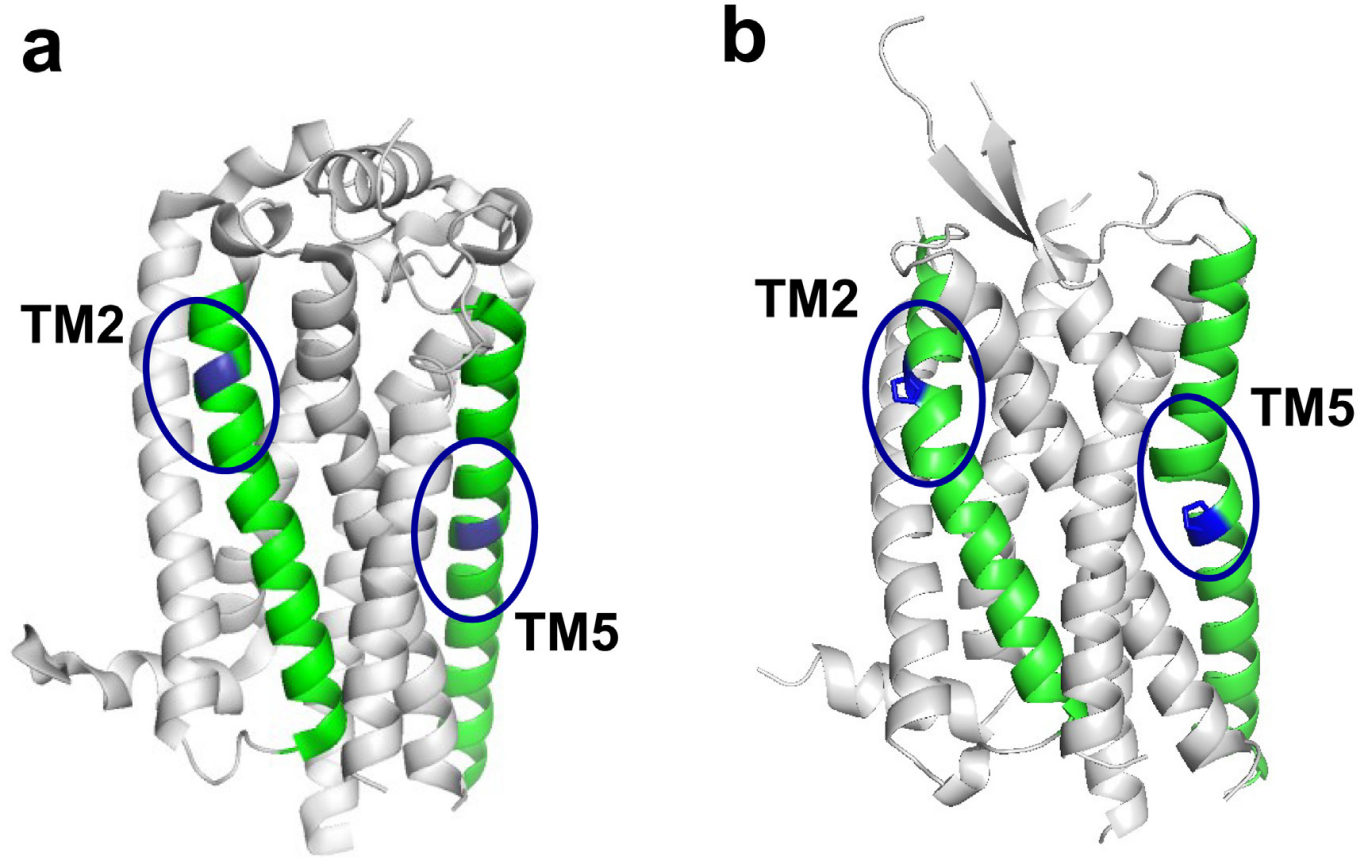
Structure of selected GPCRs. Ribbon representation of the crystal structure of (a) S1P1R (PDB # 3V2Y) and (b) OX2 (PDB # 4S0V). TM2 and TM5 are green. Positions 2.59 and 5.50 are blue. The proline residues at positions 2.59 and 5.50 in OX2 are shown as sticks. Ovals indicate the positions of the helical distortions, if any.

The GPCR crystal structures resolved to date reveals a variety of structures for TM2 and TM5 that may be related to their proline pattern. TM2 is bulged in P2.59 receptors, such as β_2_AR (β_2_ adrenergic receptor) [18] and OX2 (orexin receptor 2) [19], and in P2.60 receptors such as squid rhodopsin [20] whereas it is kinked in P2.58 receptors, such as CXCR4 (CXC chemokine receptor type 4) [21], P2Y1 (P2Y purinoceptor 1) [22] and P2Y12 (P2Y purinoceptor 12) [23]. When no proline is present in TM2, the helix may be bulged such as in bovine rhodopsin [24] and ACM (muscarinic acetylcholine receptors) [25], or straight such as in S1P1R (sphingosine 1 receptor) [26]. TM5 is bulged in receptors with a proline residue at position 5.50, such as rhodopsin [24], β_2_AR [18], OX2 [19], CXCR4 [21] and P2Y1 [22], whereas TM5 is straight in receptors with no proline in TM5, such as S1P1R [26] and P2Y12 [23]. Fig 1 represents the crystal structures of S1P1R, with straight TM2 and TM5, and of OX2, with bulged TM2 and TM5.

LGR receptors do not possess proline residues in TM2 and TM5, which prevents *a priori* prediction of the structures of these helices. However, modelling TSHR with straight or bulged TM2 and TM5 will result in a frame shift of their extracellular sides, resulting in models with different putative binding sites for small ligands. A previous study carried out by Kleinau and co-workers [27] supports the hypothesis of a straight TM5, consistent with the structures of S1P1R and P2Y12. Here, we investigate the structure of TM2 with a set of TM2 mutants. Proline residues were introduced at different positions in TM2 (2.58, 2.59 and 2.60) alone or in combination with a proline mutation at position 5.50 in TM5, then the functional properties of the mutated receptors were analyzed. Coupled with sequence analysis of the LGR subfamily and to dynamics homology modelling [28], our data support a model of TSHR with a straight TM5 and a bulged TM2.

## Materials and Methods

### Materials

DMEM/Ham’s F12 (Dulbecco’s Modified Eagle’s Medium/Ham’s F12) was purchased from PAA Laboratories (Pasching, Austria). Fetal calf serum was purchased from Biowest (Nuaille, France). Glutamine, penicillin and streptomycine were purchased from Lonza (Verviers, Belgium). Polyethylenimine (PEI) was purchased from Polysciences (Warrington, United Kingdom). The pcDNA3.1 plasmid containing the cDNA for human TSHR with a rho tag sequence at the N-terminus was kindly provided by Dr S. Costagliola (Université Libre de Bruxelles, Bruxelles, Belgium) [29]. Mouse anti-rho tag primary antibodies ORI2156 [29] were kindly provided by Dr P. Hargrave (University of Florida, Gainesville, FL). FITC-conjugated goat anti-mouse secondary antibodies were purchased from Dako (Courtaboeuf, France), and goat anti-mouse IgG secondary antibodies HRP 31430 were purchased from Thermoscientific (Waltham, USA). Luminol was purchased from Santa Cruz Technology (Dallas, USA). EndoH and PNGaseF were purchased from Promega (Fitchburg, USA). Thyrotropin from bovine pituitary (bTSH) was obtained from Sigma-Aldrich (St Louis, United-States). All other reagents were purchased from Sigma-Aldrich.

### Site-directed mutagenesis

TSHR mutants were generated by GeneCust (Dudelange, Luxembourg), from the pcDNA3.1 plasmid containing the rho tag TSHR sequence. The rho tag includes 19 amino acids from the N-terminus of rhodopsin that have been linked to the N-terminus of TSHR [29]. All the mutated plasmids were sequenced for control.

### Cell culture and transfection

HEK-293 cells were grown in DMEM/Ham's F12 medium supplemented with 10% fetal calf serum, 1% glutamine and 1% penicilline-streptomycine at 37°C in a humidified 5% CO_2_ incubator. Cells were transiently transfected in 6-well (222 222 cells/well) or 96-well (11 111 cells/well) plates with the plasmid constructs (246 ng total for 10^6^ cells) using PEI (738 ng of PEI for 10^6^ cells) and allowed for platting. The medium was changed 20 hours after transfection.

### Flow cytometry measurements

The cell surface expression was determined by flow cytometry. Two days after the transfection, cells were detached from the 6-well plates using PBS/EDTA (8mM). Suspended cells were centrifuged to discard supernatant. The following steps were performed in FACS buffer (PBS containing BSA 0.1%). Cells were incubated for 30 min with a 1:10 mouse anti-rho tag antibody, then they were washed once and incubated for 30 min with a 1:20 FITC-conjugated goat anti-mouse antibody and with propidium iodide 5 μg/ml to label damaged cells. From this part, cells were maintained at 0°C. Cells were washed and resuspended in FACS buffer. The fluorescence signal of 10^4^ cells/tube was measured. Cells transfected with empty pcDNA3.1 plasmid were used as a negative control. Flow cytometry measurements were performed on FACSCalibur^*TM*^ from BD Biosciences (Franklin Lakes, USA). Propidium iodide positive cells were excluded. Expression of mutated receptors is given as a percentage of the wild type control. FACS measurements were also performed on cells permeabilized with saponin (0.1 mg/mL).

### Determination of the glycosylation states

Glycosylation states of TSHR were analysed by Western blots. Two days after transfection, cells seeded in 6-well plates were washed with cold PBS, and lysed with SDS buffer (SDS 1%, Tris 10mM, Na Ortovanadate 1mM, Na Fluoride 10mM, antiprotease). Insoluble debris were removed by centrifugation (15 min at 13000 g). The samples were run on 8% acrylamide gel and probed with 1:10000 mouse anti-rho tag antibody. The receptors were visualized with a 1:5000 goat anti-mouse antibody and Luminol (60 seconds exposure time). The ratio of the different bands was determined with the Fujifilm Luminescent Image Analyzer LAS-3000.The glycosylation forms of the bands were determined by glycosidase treatments. Endoglycosidase H (EndoH) and Peptide-*N*-Glycosidase F (PNGaseF) were used according to the supplier’s protocol, overnight, before running. EndoH cleaves within the chitobiose core of high mannose and some hybrid oligosaccharides. PNGaseF cleaves between the innermost GlcNAc and Asn residues of high mannose, hybrid, and complex oligosaccharides N-linked to a protein.

### cAMP accumulation assay for TSH dose-response

The accumulation of intracellular cAMP was measured with the GloSensor cAMP Assay from Promega (Fitchburg, USA). The pGloSensor^*TM*^-22F cAMP plasmid was transfected simultaneously with the receptor plasmid. Two days after the transfection, cells from the 96-well plates were pre-equilibrated with GloSensor^*TM*^ cAMP Reagent for two hours. Then bTSH was added and luminescence was measured after 30 min. Cells transfected with empty pcDNA3.1 plasmid were used as a negative control. Luminescence assays were performed on Synergy 3^*TM*^ from BioTek (Vermont, USA).

### Constitutive cAMP activity of TSHR

To compare the constitutive activity of the wild type (WT) and mutated receptors for cAMP accumulation, the cells were transfected with increasing amounts of the pcDNA3.1 plasmid containing WT or mutated TSHR sequence, resulting in different cell surface expression of the receptors. The total plasmid concentration was kept constant by addition of empty pcDNA3.1 plasmid. Rolipram (25.10^−6^ ng/μl) was added 30 min before luminescence measurement of basal cAMP concentration. Then, the linear regression of the luminescence signal (basal cAMP) as a function of the surface expression of the receptor measured by flow cytometry was determined with R (www.r-project.org). We used the luminescence of the cells transfected with the empty pcDNA3.1 plasmid as the origin. For the statistical tests, the slopes of the luminescence vs the fluorescence signal of the mutated receptors were determined relatively to the WT control, set to 1, then Student’s t-tests were used for statistical analysis.

### Intracellular Ca^2+^ measurement

Two days after the transfection, cells in 96-well plate were incubated for one hour at room temperature with reduced light with Fura-2-acetoxymethyl ester (Fura-2 AM, 2μM) in HBSS buffer (CaCl_2_ 2.5mM, MgCl_2_ 1mM, Hepes 5mM et BSA 0.5%, pH = 7.3). Cells were washed twice, and then incubated for one hour with HBSS buffer before fluorescence measurement. Cells transfected with empty pcDNA3.1 plasmid were used as a negative control. The fluorescence measurements were performed with a FlexStation 3 from Molecular Devices (Silicon valley, USA). The fluorescence emitted by Fura-2 AM at 510 nm was measured upon excitation at 340 nm (Ca^2+^ bound Fura-2) and 380 nm (Ca^2+^ free Fura-2). The ratio of the fluorescence signals and the resulting AUC (area under the curve) were then calculated with GraphPad Prism (GraphPad Software, San Diego, USA).

### Data and statistical analysis

All experiments were performed in triplicates or more. For Western blots, the areas of relative density plots for each condition were extracted with ImageJ (www.imagej.net). The percentage of complex glycosylated receptors on total receptors (complex glycosylated receptors + high mannose-glycosylated receptors) for each condition was then compared to the wild type with the Student’s t test. P values less than 0.05, 0.01 and 0.001 are shown with 1 (*), 2 (**) and 3 (***) asterisks, respectively.

### Sequence analysis

The non-redundant sets of the LGR sequences from *H*. *sapiens*, *D*. *rerio*, *C*. *intestinalis*, *B*. *Floridae*, *D*. *melanogaster*, *C*. *elegans* and *N*. *vectensis* were prepared as described elsewhere [17]. Multiple sequence alignment (MSA) was carried out with Clustal X 2.1 [30] and manually refined with Genedoc [31]. The MSA is available as Supporting Information (S1 File). Bootstrapped (500 replicates) neighbor-joining (NJ) trees were built with the MEGA 5 software [32], from the alignment of the transmembrane helices. Positions in the transmembrane helices were numbered according to Ballesteros’ nomenclature [16]. N432, D474, R519, W546, P639, and P675 of TSHR were used as the reference position *n*.50 of each helix *n*, except TM5, for which Y601 of TSHR, corresponding to the highly conserved Y5.58, was used as the reference.

### Molecular modelling and dynamics simulations

The transmembrane domain of human TSHR (residues D410 to S694) was modeled with MODELLER 9v8 [33], using the crystal structure of the orexin receptor 2, OX2 (PDB # 4S0V) [19] as a template. Peptide receptors are better suited templates to model peptide/protein receptors [34]. OX2 was chosen among available structures of peptide receptors because it possesses bulges in both TM2 and TM5, and the Y5.38 and Y5.58 patterns in TM5. The insertion in the extracellular loop (ECL) 1 of TSHR was modeled as a helix based on secondary structure prediction using Jpred3 [35]. The backbone of the ECL2 hairpin was positioned in the vicinity of the K660 side chain (position 7.35) by adding distance constraints to take into account experimental data [36]. TM2 of wild type TSHR was modeled with a bulge similar to that observed in OX2 whereas TM5 was modeled with a deletion at position 5.45. Fifty models were generated and refined by simulated annealing. The models were classified using the MODELLER objective function score. The positions and nature of the violations of the top three models were analysed, to determine the “best” model for molecular dynamics simulations (MD).

MD simulations in explicit membrane environment were carried out using NAMD v2.8 MD software [37]. The “best” model was prepared for simulations in explicit membrane environment using the Charmm Gui interface [38, 39]. Models were embedded in a palmitoyl-oleoyl-phosphatidyl-choline (POPC) lipid bilayer and solvated in a TIP3P model for water molecules [40], with all atoms represented explicitly. Chloride ions were added to the system to achieve electro neutrality. The CHARMM27 (with cross-term correction for backbone dihedral angles) and CHARMM36 parameter sets were used for the protein [41, 42] and for POPC lipids [43, 44], respectively. The entire assembly was then subjected to energy minimization for 10000 steps to remove close contacts between receptor atoms and solvent or lipid layers. Equilibration of protein–membrane system was carried out with a protocol modified from that developed by Jo *et*. *al* [45]. The protocol included six interlinked equilibration steps in which harmonic restraints were gradually taken off to achieve a smooth relaxation, for a total of 1.7 ns. In the first three equilibration steps, the NVT ensemble at 310 K and time step of 1 fs were used. During the next equilibration steps and the production phase of 60 ns, the system was run at constant temperature (310 K) and pressure (1 atm), using a 2 fs time step for integration. The SHAKE algorithm was applied to all bonds involving hydrogens. The Particle Mesh Ewald method (PME) was used to calculate the electrostatic contribution to non-bonded interactions with a cutoff of 12 Å. The cutoff distance of the van der Waals interaction was 12 Å. The equilibration steps were carried out locally on a NEC 140Rb-4 server. The production steps were carried out on the E-Biothon cloud platform (www.e-biothon.fr). Trajectories were analyzed with VMD software [46]. H-bonds were measured with HBPLUS [47], Bend angles were measured using in-house script [13]. PYMOL (DeLano Scientific LLC, San Franscisco, USA) was used for figure preparation.

## Results

### Phylogenetic analysis indicates a deletion in TM5 of TSHR

**Fig 2:**
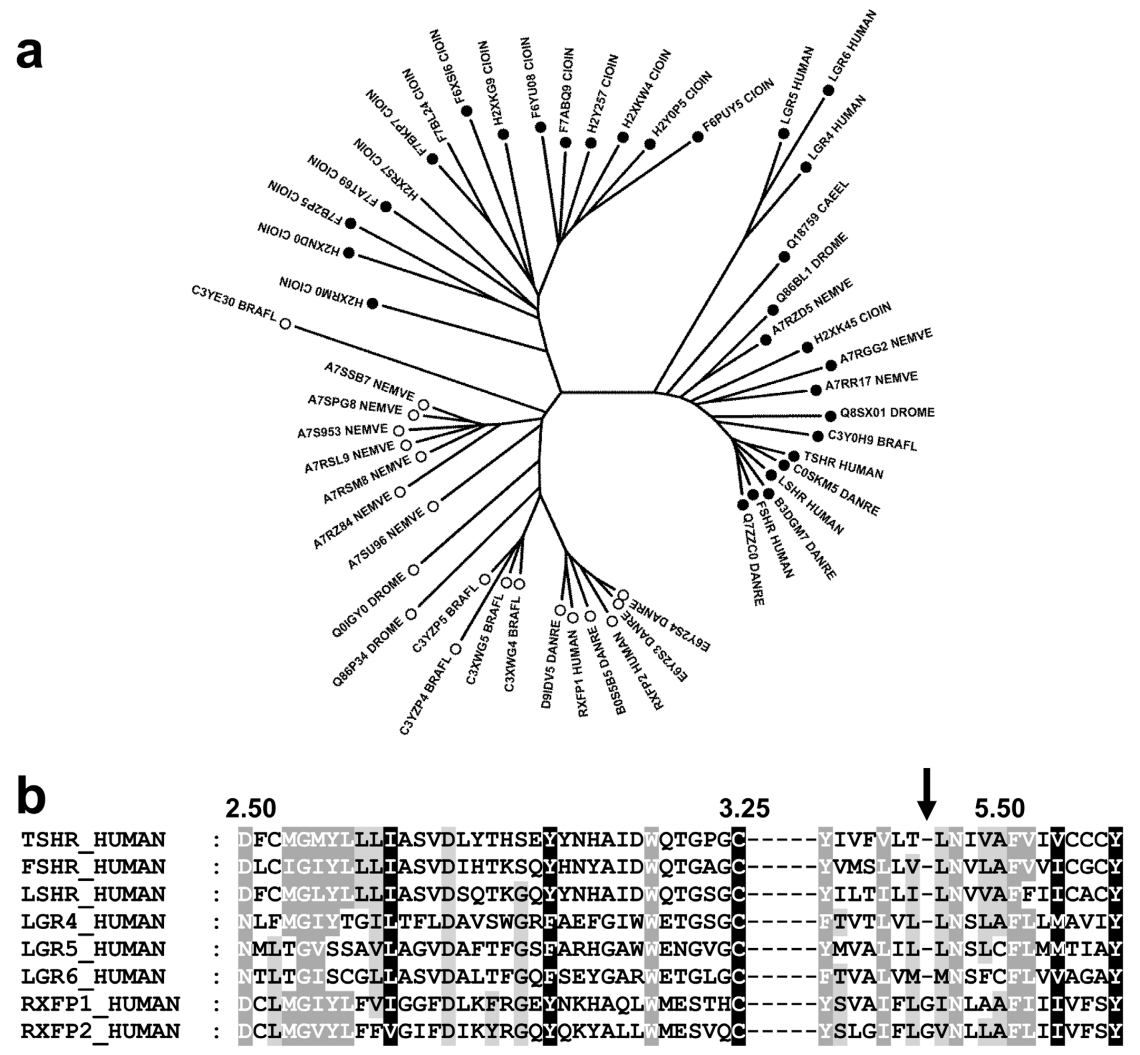
Evolution of LGR receptors. (a) NJ tree of 52 LGR receptors from seven species. The Y5.38 and Y5.39 patterns are indicated by an open and a closed symbol, respectively. (b) Sequence alignment of residues 2.50 to 3.25 and residues 5.38 to 5.58 of the human LGR receptors. The shading corresponds to the alignment of the 52 sequences. Fully conserved positions are shaded in black, partially conserved or type-conserved positions are shaded in dark grey (80% conservation) or light grey (60% conservation).

The analysis of class A GPCRs from seven species (two vertebrates: *H*. *sapiens* and *D*. *rerio*, two chordates: *C*. *intestinalis* and *B*. *Floridae*, one insect: *D*. *melanogaster*, one nematode: *C*. *elegans*, and one cnidarian: *N*. *vectensis*) led to a set of 52 non-redundant sequences of LGR receptors (sequence identities < 90%). The NJ tree based on the alignment of the transmembrane helices indicates three sub-groups (Fig 2A). The first sub-group includes receptors similar to the glycoprotein hormone receptors and to LGR4-6. The second sub-group includes receptors similar to the relaxin/insulin-like family peptide receptors 1 and 2 (RXFP1/2). These two groups are present from cnidarians to vertebrates. The third sub-group corresponds to an extension of the LGR subfamily specific to *C*. *intestinalis*. Analysis of these sequences reveals that the receptors from group 2 have a tyrosine residue at position 5.38, whereas the receptors from groups 1 and 3 have a tyrosine residue at position 5.39 (S1 File).

Comparison with other class A GPCR sequences indicates that the Y5.38 pattern is widely present in peptide and amine receptors and in opsins. Conversely, the Y5.39 pattern is present in the MEC subfamily (melanocortin, sphingosine 1 phosphate and cannabinoid receptors). The alignment of the TM5 sequences of LGR receptors with conserved N-terminal Y5.38/Y5.39 and C-terminal Y5.58 motifs requires a gap (Fig 2B for human sequences and S2 File for the entire set). The best location for the deletion is position 5.45 because it maintains hydrophobic pattern observed between the N-terminal Y5.38/Y5.39 and the conserved N5.47 residue. This phylogenetic analysis supports a model in which glycoprotein hormone receptors evolved from the deletion of one residue in the bulge of TM5 in an ancestral LGR receptor and corroborates the straight structure of TM5 in TSHR proposed by Kleinau and co-workers [27].

By contrast, TM2, TM3 and the connecting ECL1 are highly conserved, with no evidence of insertion / deletion between the highly conserved D2.50 and C3.25 residues (Fig 2B), and the alignment provides no information on the structure of TM2. In order to gain structural information on TM2, we thus engineered proline mutants in TM2 positions where proline residues may be found in class A GPCRs: 2.58, 2.59 or 2.60 [17]. We also engineered a single proline mutant at position 5.50, for comparative purpose, and double proline mutants in TM2 and TM5 to study the independence of the mutations in these two helices.

### Cell surface expression and glycosylation are impaired for most mutants except L2.59P TSHR

**Fig 3:**
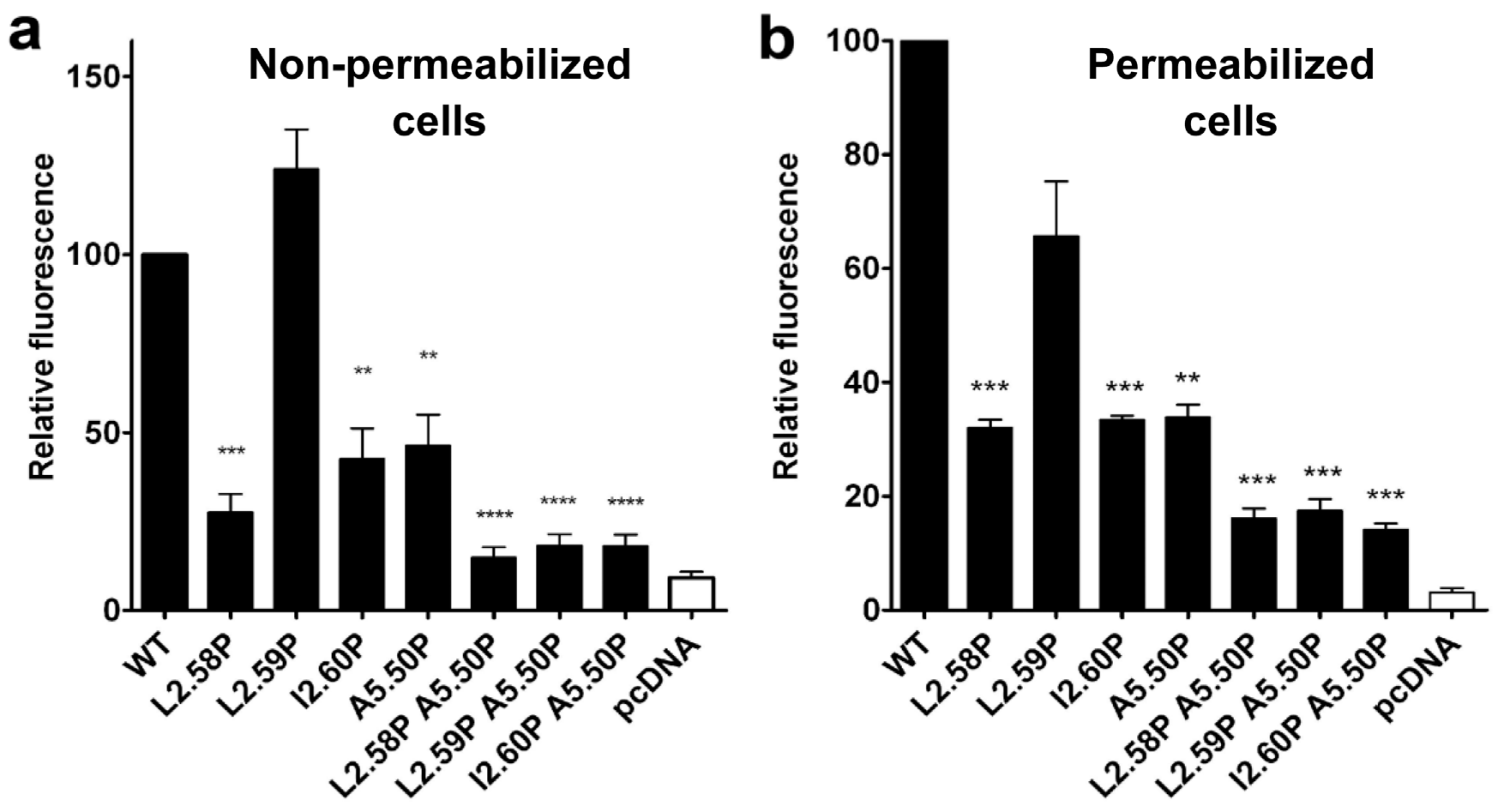
Expression of the TSHR mutants. (a) Cell surface expression and (b) total cell expression of WT and mutated TSHRs, given as the percentage of the wild type control, after transfection of HEK-293 cells by the same amount of TSHR coding pcDNA3.1 plasmid. Receptor expression was determined by flow cytometry measurements of intact (a) or permeabilized cells (b). The histograms represent the mean ± SEM of at least 3 independent experiments, each carried out in triplicates.

HEK-293 cells were transiently transfected with the pcDNA3.1 plasmid containing the sequence of WT or mutated TSH receptors. The receptor expression was measured by flow cytometry on intact and permeabilized cells (Fig 3). The L2.59 mutant had cell membrane expression (130 ± 20%) similar to WT. However the cell membrane expression decreased to about 40% of WT for the I2.60P and A5.50P mutants, to 20% of WT for the L2.58P mutant, and to less than 10% for the double mutants (Fig 3A). In permeabilized cells (Fig 3B), the amount of receptors observed were 60 ± 10% of WT for the L2.59P mutant, about 30% of WT for the L2.58P, I2.60P and A5.50P mutants and about 15% of WT for the double mutants.

**Fig 4:**
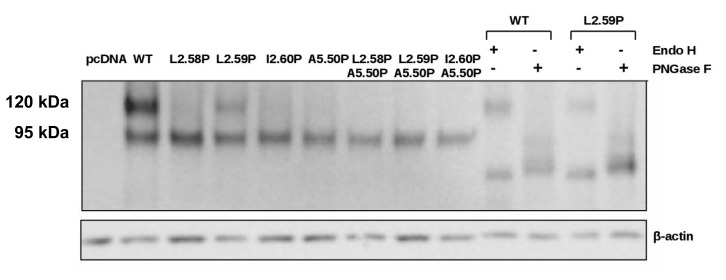
Glycosylation status of the TSHR mutants. Typical western blots of WT and mutated TSHRs, representative of three independent experiments. The identity of the bands was verified by treatment with endoH and PNGase. The band at 120 kDa corresponds to complex glycosylated receptors that are endoH resistant and PNGaseF-sensitive. The band at 95 kDa corresponds to high mannose-glycosylated receptors that are endoH and PNGaseF-sensitive.

N-glycosylation is required for cell surface expression and activity of TSHR [48, 49]. The glycosylated form(s) of WT and mutated receptors present in cells can be visualized using Western blots (Fig 4). As previously observed by others [48], WT TSHR was detected as two bands corresponding to a molecular weight of 120 and 95 kDa. Among the mutated receptors, only the L2.59P mutant presented the same two bands as WT. The glycosylation states of these forms were verified with the endoH and PNGaseF glycosidases (Fig 4). The 95 kDa form corresponds to high mannose-glycosylated receptors that are endoH and PNGaseF-sensitive. The 120 kDa form corresponds to complex glycosylated receptors that are endoH resistant and PNGaseF-sensitive. The endoH resistant 120 kDa form represented 62 ± 7% of the total amount of WT and 32 ± 3% of the L2.59P mutant. This high molecular weight form was not detectable (< 4%)) for the other mutants that were present in the cells but only as a high mannose-glycosylated 95 kDa form. Absence of complex glycosylation did not prevent some of these mutants to be addressed to the membrane (Fig 3A), in agreement with Nagayama *et al*. [48]. The low membrane expression of the L2.58P mutant cannot be attributed to the absence of complex glycosylation since this is also the case for the I2.60P and A5.50P mutants that have total cell expression similar to the L2.58P mutant (Fig 4), implying specific blockage of most L2.58P mutants at a quality control checkpoint during membrane targeting [50].

### Single mutations have differential effect on TSHR activity

**Fig 5:**
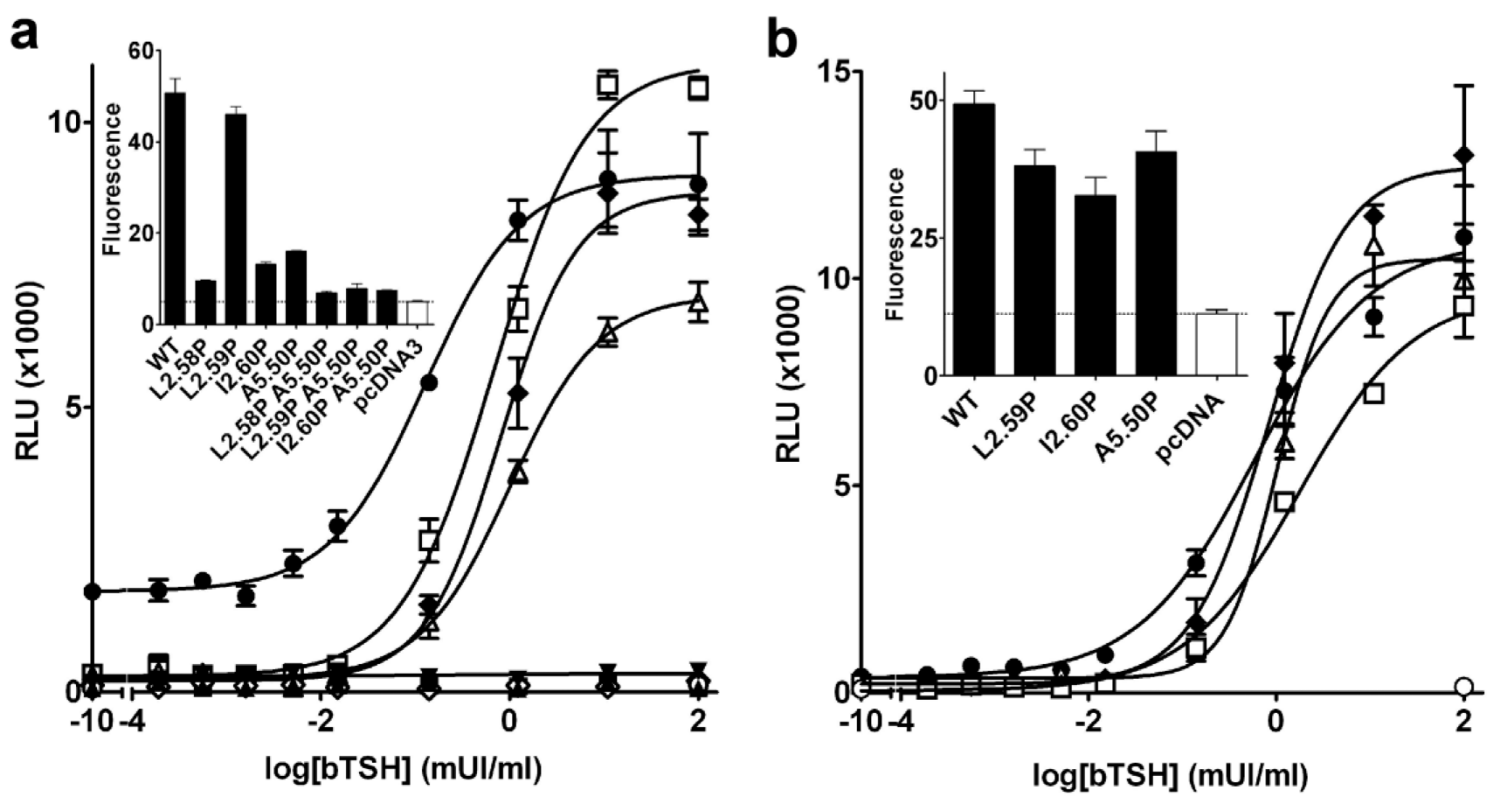
TSH induced cAMP response of TSHR mutants. Typical dose-response curves of cAMP accumulation induced by TSH on HEK-293 cells transfected with pcDNA3.1 plasmids encoding WT or mutated TSHRs. In (a), the cells were transfected with the same amount of TSHR coding plasmids, leading to the cell membrane expression shown in insert. In (b), the cells were transfected with different amounts of TSHR coding plasmids to obtain similar levels of cell surface expression, shown in insert. The total amount of pcDNA3.1 plasmids was kept constant by addition of empty plasmids. Receptor symbols: closed circles, WT; closed down triangles, L2.58P; open squares, L2.59P; open triangles, I2.60P; closed diamonds, A5.50P; closed squares, L2.58PA5.50P; closed triangles, P2.59PA5.50P; open diamonds, I2.60PA5.50P.

TSHR activates the cAMP pathway, both constitutively and upon TSH stimulation [51]. In a first assay, we measured TSH induced cAMP activity for all mutants in HEK-293 cells transfected with the same amount of TSHR coding pcDNA3.1 plasmid (Fig 5A). In this assay, the constitutive activity of WT TSHR was observed as a very high baseline, which was not observed for any mutant, including the L2.59P mutant. TSH induced a robust cAMP response for WT and the L2.59P, I2.60P and A5.50P mutants. Conversely, the L2.58P mutant and the double mutants did not induce cAMP accumulation in response to TSH. In spite of the low membrane expression of the L2.58P mutant, its constitutive activity could be observed as a baseline significantly above the baseline obtained with the plasmid control. This strongly suggests that the absence of TSH induced cAMP accumulation corresponds to an impaired mechanism of activation by bTSH. For the double mutants, the response did not differ from the plasmid control. This might be related either to impaired activation or to the very low membrane expression of these mutants. In a second assay (Fig 5B), we adjusted the amounts of the pcDNA3.1 plasmid coding for WT and single mutants to yield roughly the same level of membrane expression for the different receptors (with the exception of the L2.58P mutant due to its low membrane expression). In these conditions, the maximal activities were in the same range for the WT receptor and the L2.59P, I2.60P and A5.50P mutants (80 ± 10% of the maximal activity obtained with a 10-fold increase in the amount of the transfected plasmid containing the WT sequence). The EC50 of the mutants did not significantly differ from WT with similar levels of membrane expression (Table 1).

**Table 1:** Functional characterisation of cAMP accumulation by the TSHR mutants.

TSHR	Inducible activity^a^			Relative constitutive activity^e^
	EC50^b^	Emax^c^	CSE^d^	
**WT**	0.4 ± 0.2	80 ± 6	39 ± 4	1
**L2.58P**	-	-	-	1.7 ± 0.3
**L2.59P**	0.8 ± 0.4	70 ± 4	27 ± 3	0.23 ± 0.05**
**I2.60P**	0.8 ± 0.2*	84 ± 3	23 ± 2	0.31 ± 0.08*
**A5.50P**	0.6 ± 0.2	88 ± 6	31 ± 2	0.26 ± 0.03**

^a^ HEK-293 cells were transiently transfected with variable amounts of the pcDNA3.1 plasmid containing WT or mutated TSHRs, adjusted to yield similar cell surface expression in order to minimize the effect of the receptor concentration on the measured parameters. The WT_max_ control was obtained by transfecting HEK-293 cells with a 10 fold increase in the amount of pcDNA3.1 plasmid encoding WT TSHR. The data are given as the mean ± SEM of three independent experiments, each carried out in triplicates.

^b^ EC50 is expressed in mUI/ml. The EC50 for the WT_max_ control is 0.2 ± 0.1.

^c^ Emax values are expressed as a percentage of the WT_max_ control.

^d^ The cell surface expression (CSE) was quantified by flow cytometry measurements. CSE levels are expressed as a percentage of the WT_max_ control

^e^ The relative constitutive activity of the TSHR mutants was estimated from the ratio of the slopes in the linear regression of the basal cAMP level as a function of the cell surface expression for mutated and WT receptors. The constitutive activity of the WT control is set to 1. The data are given as the mean ± SEM of three independent experiments, each carried out in triplicates.

**Fig 6:**
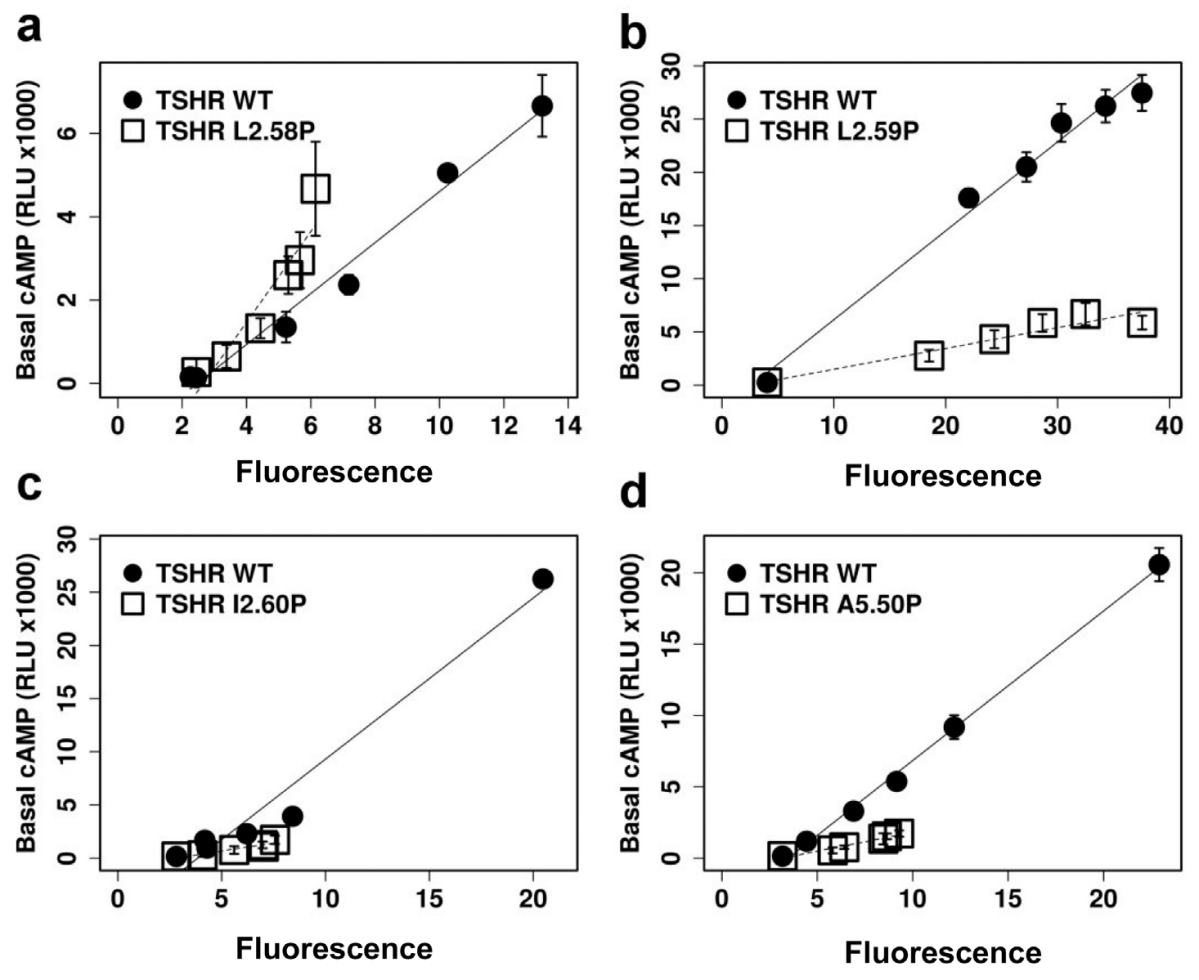
Constitutive cAMP activity of TSHR mutants. Typical measurements of the constitutive activity of the L2.58P (a), L2.59P (b), I2.60P (c) and A5.50P (d) TSHRs. In each panel, the cAMP accumulation obtained in the presence of the mutated and WT TSHR is indicated by open and closed symbols, respectively.

We measured the constitutive activity of WT and mutated receptors by the basal level of cAMP observed at different levels of receptor membrane expression (Fig 6 and Table 1). For each mutant, a control with WT was made in the same experimental set. The ratio of the slopes gives an estimate of the relative constitutive activities. A 3–4 fold decrease in the constitutive activity was observed for the L2.59P, I2.60P and A5.50P mutants. This was not the case for the L2.58P mutant whose constitutive activity was at least as high as that observed for WT receptor (Table 1). The differences between the A5.50P mutant data reported by Kleinau *et al*. [27] and here might result from the higher level of cell membrane expression measured here (40% vs 6%), in link with different antibodies (an anti-rho tag vs conformational antibody). In either case, the data indicates that the substitution of a proline at position 5.50 does not completely disrupt the functionality of TSHR.

**Fig 7:**
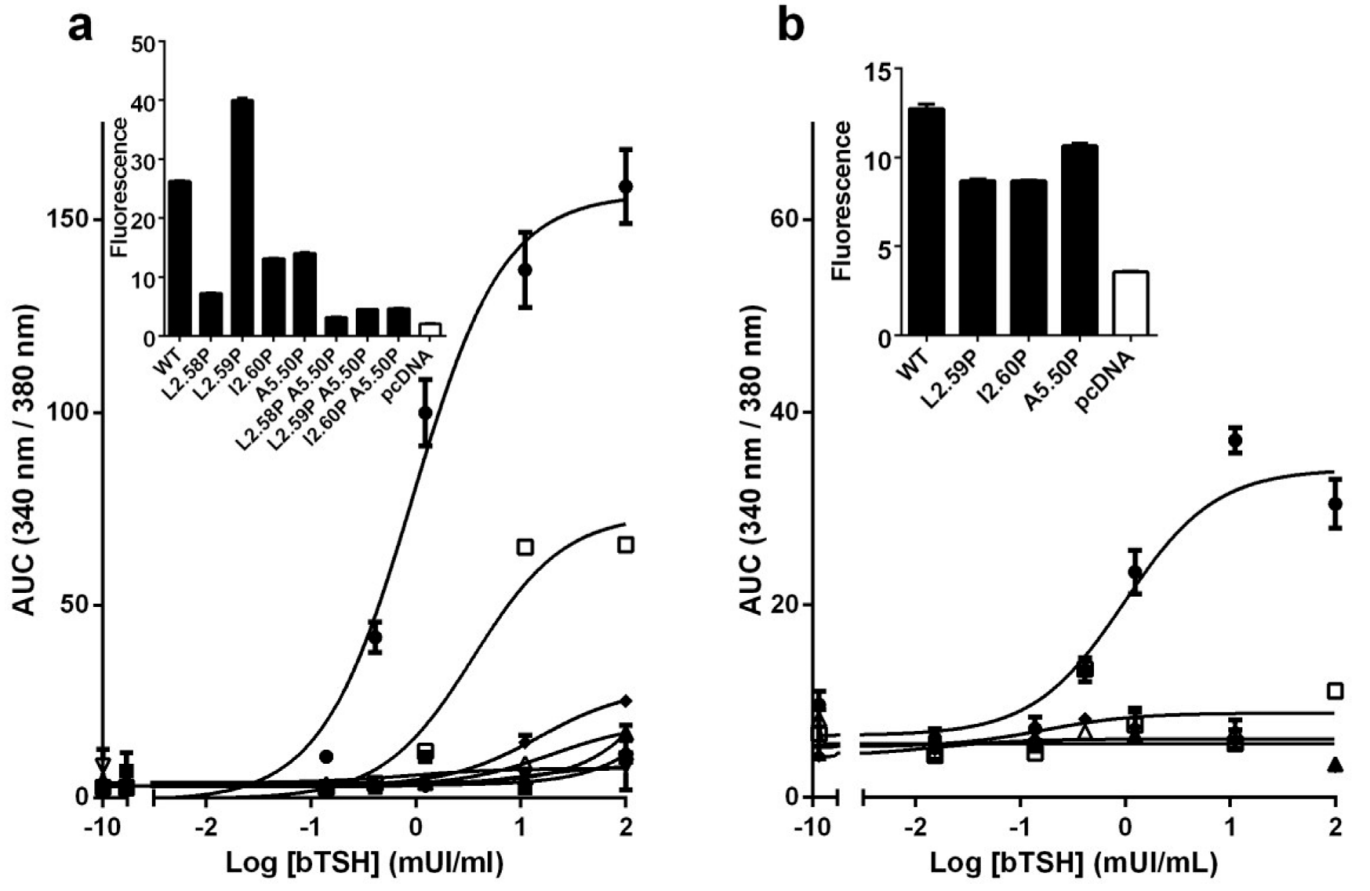
TSH induced Ca^2+^ response of TSHR mutants. Typical dose-response curves of Ca^2+^ accumulation induced by TSH on HEK-293 cells transfected with pcDNA3.1 plasmids encoding WT or mutated TSHR. In (a), the cells were transfected with the same amount of TSHR coding plasmids, yielding the membrane expression shown in insert. In (b), the cells were transfected with different amounts of TSHR coding plasmids to obtain similar levels of cell surface expression, shown in insert. The total amount of pcDNA3.1 plasmids was kept constant by addition of empty plasmids. Receptor symbols: closed circles, WT; closed down triangles, L2.58P; open squares, L2.59P; open triangles, I2.60P; closed diamonds, A5.50P; closed squares, L2.58PA5.50P; closed triangles, P2.59PA5.50P; open diamonds, I2.60PA5.50P.

TSHR activates the Ca^2+^ pathway in response to TSH [52]. To determine which mutants can activate this pathway, in a first assay, we measured the Ca^2+^ accumulation in response to TSH for all mutants, in cells transfected with the same maximal amount of pcDNA3.1 plasmid (Fig 7A). Compared to the cAMP pathway with EC50 around 0.2 mUI/ml, the activation of the Ca^2+^ pathway by the wild type receptor required a five-fold higher concentration of TSH (Fig 7A). Activation to about half the maximum activity of WT was observed only for the highly expressed L2.59 mutant. No significant activation could be detected for the other mutants. Subsequently, in a second assay, we measured the Ca^2+^ accumulation in response to TSH for the WT control and single mutants (except L2.58P) with comparable cell surface expression (Fig 7B). In these conditions, we did not observe significant Ca^2+^ accumulation in response to TSH for any of the mutants.

### Dynamic homology modelling supports a bulged TM2 and a straight TM5

**Fig 8:**
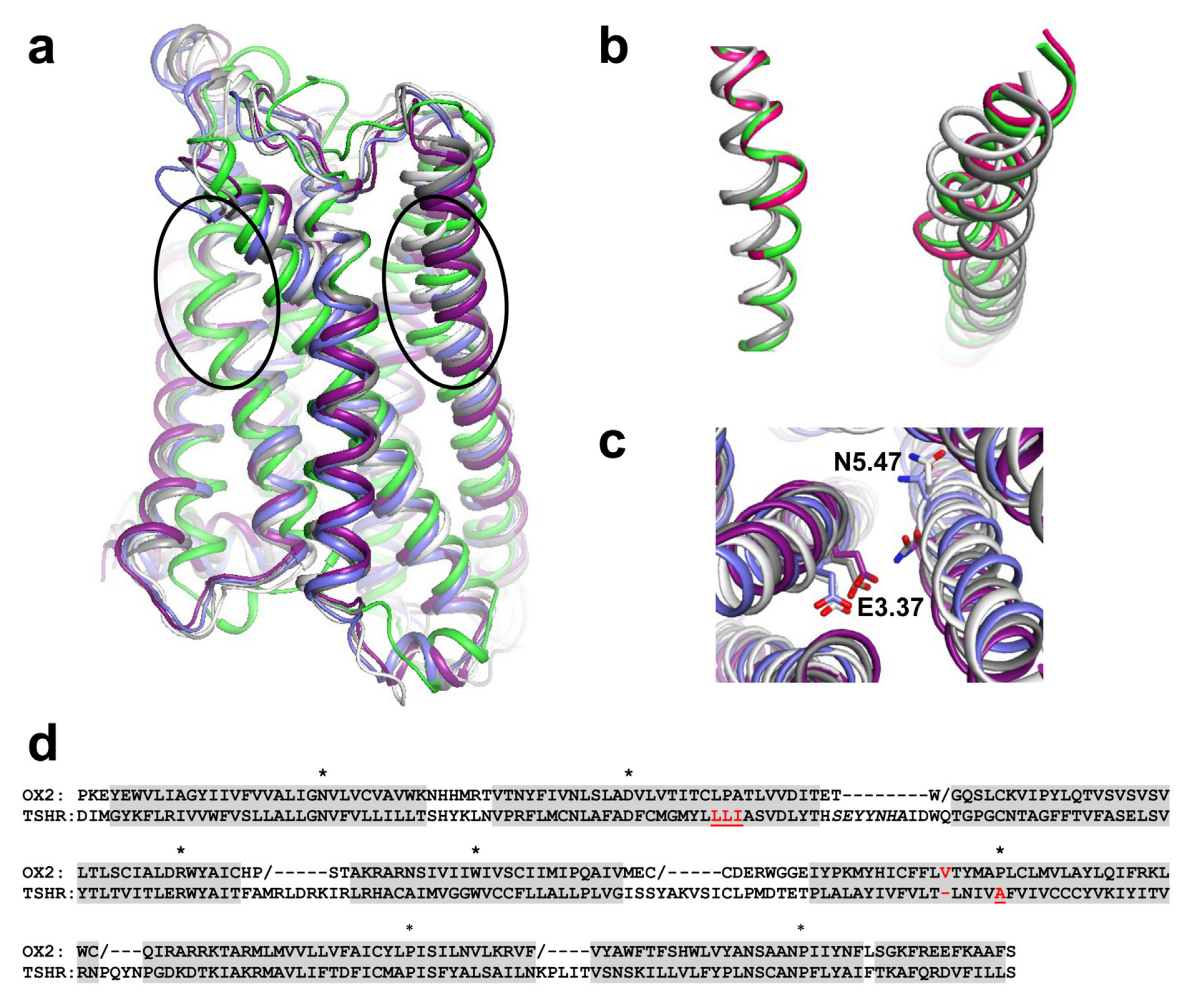
Dynamic modelling of the TSHR transmembrane domain. **(a)** Cartoon representation of TSHR transmembrane domain, obtained by homology modelling with the OX2 template, before and after simulations. The initial model is green. The snapshots obtained after 10, 20, 40 and 60 ns MD simulations are white, slate, grey and purple, respectively. The ovals indicate the positions of the distortions in TM2 and TM5. (b) TM2 (left) and TM5 (right) of the OX2 template (magenta) superimposed with the initial TSHR model (green) and with the snapshots obtained after 10 and 40 ns MD simulations (white and grey, respectively). (c) Close-up view of the relative orientation of the E3.37 and N5.47 side chains in the snapshots obtained after 10, 20, 40 and 60 ns MD simulations. (d) Sequence alignment between OX2 and TSHR used for homology modelling. The / symbol in the OX2 sequence indicates interruption of the sequence due to either missing residues in the crystal structure or residues not used for structural constraints in the modelling procedure. Helices are indicated by grey shading. The residues modeled with helical constraints in ECL1 are in italic. The positions of the anchors are indicated by stars. The position of the deletion in TM5 of OX2 is red. The positions of the proline substitutions in TSHR are red and underlined.

In our set of TSHR mutants, the substitution of a proline residue at position 2.59 does not impair complex glycosylation and membrane expression, indicating that this mutation is less deleterious for receptor folding and/or processing than proline substitution at positions 2.58 and 2.60. This is consistent with the favoured positioning of proline at position 2.59 in bulged TM2 [17]. Our data thus supports a model for the transmembrane domain of TSHR with bulged TM2 and “unbulged” TM5. However, whether TM5 is straight or maintains a kinked structure could not be determined from experimental data. We thus modeled the transmembrane domain of TSHR with a kinked TM5 and assessed the stability of this structure by molecular dynamics simulations. This procedure is an efficient method to validate homology models [28]. The modelling protocol maintained the bulged structure of TM2 present in the OX2 template, but forced the deletion of one residue at position 5.45 in the TM5 bulge of OX2 (see alignment in Fig 8). As a result, the initial TSHR model presented a marked kink in TM5 with a bend angle of 26°, similar to the bend angle of the template. Then we performed MD simulations in an explicit membrane environment. The initial model and relevant snapshots are available as Supporting Information (S3 File).

**Fig 9:**
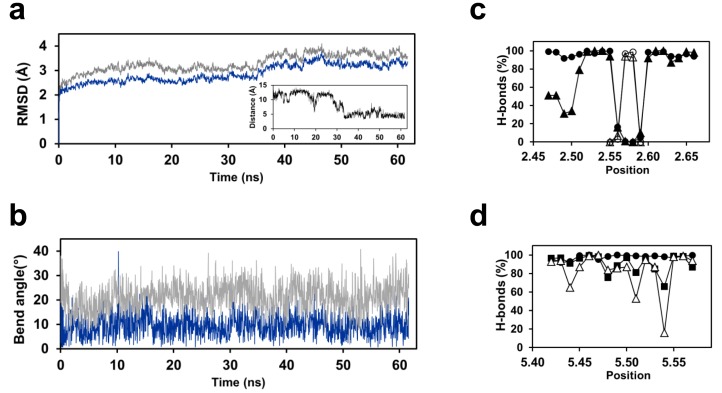
Analysis of the MD simulations of the TSHR transmembrane domain. (a) Time evolution of the root mean squared deviations (RMSD) of the Cα atoms of the TSHR transmembrane domain (grey) or transmembrane helices only (blue). The insert shows the evolution of the distance between the Cδ atom of E3.37 and the Nδ1 atom of N4.47; (b) Time evolution of the bend angle of TM2 (grey) and TM5 (blue); (c) Backbone *i* to *i*-4 (closed symbols) and *i* to *i*-5 (open symbols) H-bonds in TM2. The percent of H-bonds was measured during the equilibration phase (triangles) and the production run (circles); (d) Backbone *i* to *i*-4 H-bonds in TM5 during the equilibration phase (triangles), the 35 first ns (squares) and the 25 last ns (circles) of the production run.

Fast reorganisation of the TSHR transmembrane domain with RMSD of about 2.0 Å occurred in the first 100 ps of the equilibration phase, and was followed by slower reorganisation in the next 10 ns (Fig 9A). A first plateau around 2.5 Å was stable up to 35 ns, and then a transition led to a second plateau around 3 Å. The transition was concomitant with rotameric reorientation of the E3.37 and N5.47 side chains that led to H-bond interactions between these two residues (Fig 8C). A major reorganisation of TM5 occurred at the very beginning of the equilibration phase. During the minimisation steps, the bend angle of TM5 decreased from 26° to about 10° and resulted in the straightening of the helix. Thereafter, TM5 was stable during the remainder of the simulations, with an angle of 9° ± 5° between the N- and C-termini (Fig 9B). Nevertheless, during the equilibrium phase, the *i* to *i*-4 H-bonding pattern was not regular (Fig 9D). During the first plateau, some irregularities were still observed, whereas, during the second plateau, the helical pattern was very stable, suggesting that the interaction between the side chains of E3.37 and N5.47 stabilizes the structure of TM5. The initial bend angle of TM2 was 33°. It decreased during the equilibrium phase, along with a reorganisation of the helix (Fig 8B), then remained stable at 22° ± 6° (Fig 9B). The backbone *i* to *i*-4 H-bond pattern of TM2 was irregular during the equilibration phase then remained stable during the 60 ns production run (Fig 9C). Due to the presence of the bulge, the *i* to *i*-4 H-bonds were absent from positions 2.56 to 2.59, whereas two *i* to *i*-5 H-bonds involving positions 2.57 and 2.58 stabilized the helical distortion.

## Discussion

A variety of TM2 and TM5 structures are observed in crystal structures of class A GPCRs and are usually related to the presence of specific proline patterns [11]. However, in the absence of proline in these helices, no prediction can be done *a priori*, since kinks or bulges may be present in α-helices in the absence of proline [11, 53]. Our previous analysis of helical distortions indicates that bulges are stabilized by the presence of a proline residue that can be positioned at either one of two consecutive positions [12, 13]. For TM2 in class A GPCRs, these two positions correspond to 2.59 and 2.60 with a marked preference for 2.59, whereas the P2.58 pattern is not compatible with a bulge [17].

Proline substitutions are not easily accommodated in proteins, except when they induce only local structural alteration [53]. With the exception of constitutive activity (see below), the experimental results obtained with the proline substitutions in TM2 of TSHR indicate that the structural and/or functional impairment ranges in the following order: WT < P2.59 < P2.60 << P2.58. The tolerance for position 2.59 supports the hypothesis that the TM2 distortion in TSHR results from a vestigial proline at position 2.59 later removed during evolution and thus corresponds to a bulge. This hypothesis is corroborated by such a vestigial P2.59 proline in the closely related follitropin receptor from *M*. *auratus* (Uniprot # Q6R6L8). Moreover, the MD simulations of the TSHR model suggest that position 2.59 is not involved in either *i* to *i*-4 or *i* to *i*-5 backbone H-bonds. This is not the case for position 2.60 involved in *i* to *i*-4 H-bonds. This difference in H-bonding is consistent with the observed preference of TSHR for proline substitution at position 2.59 rather than 2.60, since proline cannot be engaged in H-bond with preceding carbonyl groups.

Concerning TM5, the evolutionary analysis carried out in this study strongly supports a deletion of one residue in TM5, consistent with the straight structure previously proposed by Kleinau *et al*., based on constitutive activity of L5.44A mutant [27]. In both studies, the data indicates that the substitution of a proline at position 5.50 does not completely disrupt the functionality of TSHR. This might be related to the external position of TM5 and the absence of steric constraints allowing local conformational changes to adapt to the mutation.

How does the proposed structure of the TSHR transmembrane domain help rationalize the effect of the mutations on the receptor functionality? The activation of TSHR depends on a switch involving D6.44 in TM6 and N7.49 in TM7 [54, 55]. The high constitutive cAMP activity of WT TSHR indicates a low activation energy barrier between the active and inactive states [56] and consequently it is very sensitive to mutations [57]. Position 2.46 has an important role in the equilibrium between active and inactive states, since the L2.46A mutation increases the constitutive activity of TSHR by a factor of 13 [55]. The marked decrease in the constitutive activity of the L2.59P and L2.60P mutants (Fig 6 and Table 1) suggests an indirect effect of these substitutions on the switch that might occur *via* L2.46. The decrease in the constitutive activity of the A5.50P mutation might result from an effect on the E3.37—N5.47 H-bond and, in turn, on the D6.44—N7.49 switch since the distance between the Cα atoms of N5.47 and D6.44 is only 9 Å. Alternatively, the proline mutations might hinder the protein flexibility necessary for activation [56].

By contrast with the other mutants, the L2.58P mutant displays a robust constitutive activity and disruption of inducible activity (Figs 5 and 6). A spontaneously occurring L2.57P mutation presents the same phenotype [58]. This suggests that a part of these receptors might be in an active-like state, and, consequently, would be unable to elicit further response to TSH. A bulged structure similar to that observed in TM2 of GPCRs is not possible with a proline residue at position 2.58 or 2.57 [12, 13, 17]. The P2.58 and P2.57 mutations might thus induce either a reorientation of TM2 or a kink with a frameshift in the extracellular half of TM2 [17], modifying the interaction between the extracellular loops, the hinge region and the ectodomain. The ectodomain acts as an inverse agonist whose inhibitory effect is released by hormone binding [29]. The P2.58 mutation might thus either hinder the inhibitory effect of the ectodomain or alter the D6.44 –N7.49 switch in the mutants able to reach the cytoplasmic membrane.

Upon thyrotropin binding, TSHR activates both Gs and Gq/11 pathways [59, 60]. Ca^2+^ accumulation results mainly from the Gq/11 pathway [61]. However, this pathway is not the primary signaling pathway and requires higher TSH concentrations [62], in agreement with our results (Figs 5 and 7). Numerous mutations that affect the Gq but not the Gs pathway have been reported [63–65] and are consistent with a reduced affinity of TSHR for Gq as compared to Gs. We observed a similar effect with proline mutants, since for the same level of expression, none of the mutants was able to induce significant calcium mobilisation (Fig 7). An elegant mechanism proposed by Allen *et al*. to rationalise the difference between Gs and Gq activation relies on the negative cooperativity of TSH binding to the TSHR dimer [62]. In this model, the simultaneous occupancy of both G protein binding sites of a TSHR dimer by Gs and Gq would be necessary for Gq activation. A recent study on TSHR dimerization interface suggests a broad interface involving TM1 and TM2 on one protomer and TM4 and TM5 on the second protomer [66]. The position of the proline mutations suggests that they might alter the dimerization interface and, in turn, impair Gq activation. Other explanations that are not mutually exclusive would be differential effects of the proline mutations on the affinity of TSHR for Gs and Gq or an effect of the glycosylation state of TSHR on Gq activation.

Finally, very low membrane and total expression of the double mutants (Fig 3) indicates impaired and/or slower folding that prevents them from fulfilling quality control for export and membrane targeting. The synergetic effect of mutations in TM2 and TM5 is striking with the L2.59PA5.50P mutant. Albeit the L2.59P and A5.50P single mutations do not prevent TSHR from eliciting a robust cAMP response, the L2.59PA5.50P double mutation is more deleterious than the additive effects of the single mutations. This suggests that conformational flexibility allowing the receptor to adapt to a mutation is not limited to the vicinity of the mutated residue but also involves long range residues.

## Conclusions

Experimental data and bioinformatics analysis carried out here strongly supports the assumption that TSHR and related glycoprotein hormone receptors share a common transmembrane fold, characterized by a bulged TM2 and a straight TM5, with no similar crystal structure reported yet. In the absence of crystal structure, this analysis provides a better understanding of structural determinants of the transmembrane domain of TSHR and related receptors, which should be advantageous in the design of drugs that are targeted towards the internal binding site of these receptors. The strategy of proline substitution in TM2 and TM5 appears to be a useful method to help model these helices in GPCRs when no proline can guide modelling and no closely related template is available. Modelling these helices with kinked, straight or bulged conformations alters the residues facing the internal cavity of the receptors. The correct structural conformation of these helices is thus of crucial importance for drug design.

## Supporting Information

S1 File Sequence alignment of the LGR receptors in fasta format. The sequence set includes 52 LGR receptors from *H*. *sapiens*, *D*. *rerio*, *C*. *intestinalis*, *B*. *Floridae*, *D*. *melanogaster*, *C*. *elegans and N*. *vectensis*. The sequence names correspond to the Uniprot identifiers (entry names).
(FASTA)

S2 File Sequence alignment of the LGR receptors. Residues 2.50 to 3.25 and residues 5.38 to 5.58 of the 52 LGR receptors from *H*. *sapiens*, *D*. *rerio*, *C*. *intestinalis*, *B*. *Floridae*, *D*. *melanogaster*, *C*. *elegans and N*. *vectensis* were aligned. A gap was introduced at position 5.45 to maintain a Y/F motif at the N-terminus of TM5. Fully conserved positions are shaded in black, partially conserved or type-conserved positions are shaded in dark grey (80% conservation) or light grey (60% conservation). The sequence names correspond to the Uniprot identifiers (entry names).
(PDF)

S3 File PDB file including the initial model of TSHR and relevant snapshots. The snapshots are superimposed on the initial model. Frame 1: initial model, frames 2 to 5: snapshots obtained after 10, 20, 40 and 60 ns MD simulations.
(PDB)

## Abbreviations

TSH: Thyrotropin
GPCR: G protein-coupled receptor
LGR: leucine-rich repeat containing GPCR
TM: transmembrane
TM*i*: transmembrane helix *i*
ECL: extracellular loop
WT: wild type
MSA: multiple sequence alignment
NJ: neighbour-joining
MD: molecular dynamics
DOPC: palmitoyl-oleoyl-phosphatidyl-choline
SEM: Standard error of the mean
RMSD: root mean squared deviations
AUC: area under curve
