# Supplementary material for: Molecular Insights into the Transmembrane Domain of the Thyrotropin Receptor
Source: PLoS One. 2015 Nov 6;10(11):e0142250. doi: 10.1371/journal.pone.0142250 (PMC4636318; doi:10.1371/journal.pone.0142250)
Supplement: S2 File — Residues 2.50 to 3.25 and residues 5.38 to 5.58 of the 52 LGR receptors from H. sapiens, D. rerio, C. intestinalis, B. Floridae, D. melanogaster, C. elegans and N. vectensis were aligned. A gap was introduced at position 5.45 to maintain a Y/F motif at the N-terminus of TM5. Fully conserved positions are shaded in black, partially conserved or type-conserved positions are shaded in dark grey (80% conservation) or light grey (60% conservation). The sequence names correspond to the Uniprot identifiers (entry names). (PDF) [file pone.0142250.s002.pdf]

S2 Align. Sequence alignment of the LGR receptors.

```
FSHR_HUMAN   : DLCIGIYLLLIASVDIHTKSQYHNYAIDWQTGAGC-----YVMSLLV-LNVLAFVVICGCY
LSHR_HUMAN   : DFCMGLYLLLIASVDSQTKGQYYNHAIDWQTGSGC-----YILTILI-LNVVAFFIICACY
TSHR_HUMAN   : DFCMGMYLLLIASVDLYTHSEYYNHAIDWQTGPGC-----YIVFVLT-LNIVAFVIVCCCY
LGR4_HUMAN   : NLFMGIYTGILTFLDAVSWGRFAEFGIWWETGSGC-----FTVTLVL-LNSLAFLLMAVIY
LGR5_HUMAN   : NMLTGVSSAVLAGVDAFTFGSFARHGAWWENGVGC-----YMVALIL-LNSLCFLMMTIAY
LGR6_HUMAN   : NTLTGISCGLLASVDALTFGQFSEYGARWETGLGC-----FTVALVM-MNSFCFLVVAGAY
B3DGM7_DANRE : DLCIGIYLLMIATVDLRTRGHYSHHAIEWQTGAGC-----YVILLLL-FNVGAFLVICGCY
Q7ZZC0_DANRE : DLCMGIYLLLIAAVDIHTQSRYYNYGIDWQTGAGC-----YVVLLLL-LNAAAFLVVCVCY
C0SKM5_DANRE : DLCMGIYLLLIASVDLHTQSEYYNHAIDWQTGPGC-----YIICVLV-LNILAFIVICVCY
Q86BL1_DROME : DFFMGIYLGILAIVDAATLGEFRMFAIPWQMSVLC-----YVISLMF-INGCAFLTLMGCY
Q8SX01_DROME : DLCLGLYLLLVACFDAHSMGEYFNFAYDWQYGLGC-----YLIAILG-SNGVAFSIIAVCY
C3Y0H9_BRAFL : DFCLGFYLLMLAAADIYSSHSYYTWAIVWQTGGGC-----YVATTMI-ISVLAFGLICGCY
Q18759_CAEEL : DMVTGIYLAVLAIADAKMSDEYYRHAVWWQTGWGC-----YLIFGLS-FNFLAFAAMALSY
F6YU08_CIOIN : DILMGLFLLIICIKGVHFSGSYCYHDIAWRSSTTC-----YSTFIVT-FNFILFMFIFVSY
F7BKP7_CIOIN : DFLMGVYLAVVAGYSVSFSGKYCFEDEGWRTGTTC-----FAIVIVS-VNFLLFLFIAGSY
F7ABQ9_CIOIN : DLLMGVYLLSIGIKGSLYSGIYCYHDIEWRSSPLC-----YSMAIIT-INFVLFLYMLVGY
H2XKW4_CIOIN : DFLMGIYLITISIKGVHFSGSYCYNDLEWRTSKLC-----YSLFLIS-LNFTMFMYILVSY
F7AT69_CIOIN : DLLMGVFLIALGIRSASTSGNYCLIDHSWRSGTPC-----FSLTVTI-TNFILCLYIVGAY
H2XRM0_CIOIN : DLLMGIYLLMIAVVDSVHQVEFFAFSHIWRHSGFC-----FSLFVII-FNLVAFFAMVTSY
H2Y0P5_CIOIN : DFLMSVYLLAVSIKGLQFSSRYCYHDQEWRSSQLC-----YSTFLIT-LNFALFLYMVFSY
H2Y257_CIOIN : DLLMGVYLLSISIQGAIFSGRYCYHDMEWRSGTLC-----YSTSIII-MNFVLFIYIMVSY
F6PUY5_CIOIN : GFLMGIYLIAIAIKAAEFSKLYCYFDTDWRTSDTC-----YTTFLIT-LNTCLFIYIVACY
H2XND0_CIOIN : DLLIGVYLILLGGTGAFYSGTYCANKLTWLSSSLC-----FTFLLLS-LNLAAFIFVAASY
F7B2P5_CIOIN : DLIMGVYLIVLGSMGIKYGGKFCAHAKHWMGSGTC-----YPIVTAA-FNFTAFVFVASSY
F6XSI6_CIOIN : DGLMGVYLIIIAIMSVQFSGKYCYMDKIWRSGQLC-----FTVTVIT-FNFAAFIMMVLFY
H2XKG9_CIOIN : DGLMGIYLIILGAKGLQFSGNYCRFDKSWRTSSQC-----YSVIIIL-FNFFMFVYICVAY
H2XK45_CIOIN : DFFMGIYLFTLTAMDSRSSGNYYKFGVEWQTGGGC-----YVAVLLS-LDICAFMVVVGSY
A7RGG2_NEMVE : DFCLGLYLFILTCVSMDTHGLYHNYVRRWQYGAGC-----YVAFLLF-LNGAAFAFVLYLY
A7RR17_NEMVE : NFFMGIYLGMIATADACTYGEYQNHARAWQFGTGC-----YVTFLLI-TNGIAFLSVLFCY
A7RZD5_NEMVE : DMCMGLYLGLLAIVDASTIGDFLHHGVEWQLSTGC-----FVTFTMV-LNCAAFLVIFGCY
RXFP1_HUMAN  : DCLMGIYLFVIGGFDLKFRGEYNKHAQLWMESTHC-----YSVAIFLGINLAAFIIIVFSY
RXFP2_HUMAN  : DCLMGVYLFFVGIFDIKYRGQYQKYALLWMESVQC-----YSLGIFLGVNLLAFLIIVFSY
B0S5B5_DANRE : DCLMGVYLLFIGAFDIKYCGEYNRHAQIWMESLSC-----YSTAIFLGLNLLAFVMIVFSY
E6Y2S4_DANRE : DCLMGVYLFFVGIFDVKFRGEYNKNAKVWMESLEC-----YSTGIFLGLNLLAFLIIVVSY
E6Y2S3_DANRE : DCLMGVYLFFLGIFDVKFRGEYNRNALIWMDSVEC-----YSTGIFLGLNLVAFLVIVISY
D9IDV5_DANRE : DGLMGVYLFMIGAYDLKFRGEYNRHAQAGMDSEAC-----YSIVIFLGLNLVAFLIIVLSY
Q0IGY0_DROME : DFLMGIYLTLIACADISFRGEYIKYEETWRHSGVC-----YSALLFILVNTLSLIFILFSY
Q86P34_DROME : DMLMGFYLVTIGVQDYRYRNEYYKVVLDWITSWQC-----YSAFVFLGVNLLLLVMIAMLY
C3YZP4_BRAFL : DLVMGVYLIIIGTKDVVYRGIYNRHALEWKESFGC-----YSAFIFLGVNFTSLLIILSAY
C3XWG5_BRAFL : DLIMGVYLLIIGTKDVMLRGVYNQHAEEWKTGYGC-----YSAFVFIGINFSSLLVIMAAY
C3XWG4_BRAFL : DFIMGVYLLVIATKDMTFRGVYHRHTHAWTNSLGC-----YSAFIFLGVNFVSVLVIGAAY
C3YZP5_BRAFL : DLIMGIYLLIIGSKDHVYRDVYNQYAQEWKQSYSC-----YSAFIFLGINFTSLTVILFAY
C3YE30_BRAFL : DAIMGVYCIIIASADEHYRGRYIENAEAWKQGGLC-----YSAFLFIIFNFCASVMLIVYY
H2XR57_CIOIN : DFLMGVALLIIAIKSAQFSGQYCWRDLLWRSSTTC-----EISAVIMSFNFITLLYISAAY
F7BL24_CIOIN : DFLMGVYLICIAIYSAVFSGRYCYEDEKWRTGSSC-----RISITIVSINFFVFVFIALAY
A7RSL9_NEMVE : DFMMGLYLLIIAFKDVQWQGEYFKHDLSWRVSGLC-----YSVSFFIGLNFIAFMFILLAY
A7RSM8_NEMVE : DFLMGVYMLLIAIKDVEYQGEYFKHDIEWRSGRLC-----YSVAIFVVLNFVSFVFILVAY
A7RZ84_NEMVE : DLLMGVYLWIIAIQGVRWTGEYFRYDVQWRTGMLC-----YSFAIFIVFNSAAFLFILVAY
A7S953_NEMVE : DWLMGCYLIIIAIKDLEWRGEYFKHDVAWRSSRLC-----YSVTLFIGLNAVAFSFILIAY
A7SPG8_NEMVE : DLLMGVYLMIIAITDAKWQGEYFKHDVTWRAGIGC-----YSVAFFIGLNSIAFIFILVAY
A7SSB7_NEMVE : DLLMGVYLLLIAIMDTKWQGEYFKHDIEWRSGIGC-----YAVVIFIGVNLVAFMFMLFAY
A7SU96_NEMVE : DFMMGVYMVVIGSVDKYYRGKYFIYNEEWKRSHLC-----YSVFVFLVLNFISFMAIFILY
```

Sequence alignment of residues 2.50 to 3.25 and residues 5.38 to 5.58 of the 52 LGR receptors from *H. sapiens*, *D. rerio*, *C. intestinalis*, *B. Floridae*, *D. melanogaster*, *C. elegans and N. vectensis*. Fully conserved positions are shaded in black, partially conserved or type-conserved positions are shaded in dark grey (80% conservation) or light grey (60% conservation). The sequence names correspond to the Uniprot identifiers (entry names).
